# Comprehensive assessment of facial paralysis based on facial animation units

**DOI:** 10.1371/journal.pone.0277297

**Published:** 2022-12-14

**Authors:** Amira Gaber, Mona F. Taher, Manal Abdel Wahed, Nevin Mohieldin Shalaby, Sarah Gaber

**Affiliations:** 1 Faculty of Engineering, Systems and Biomedical Engineering Department, Cairo University, Giza, Egypt; 2 Faculty of Medicine, Neurology Department, Cairo University, Giza, Egypt; 3 Faculty of Physical Therapy, Department of Neuromuscular Disorder and Its Surgery, Cairo University, Giza, Egypt; Jadavpur University, INDIA

## Abstract

Quantitative grading and classification of the severity of facial paralysis (FP) are important for selecting the treatment plan and detecting subtle improvement that cannot be detected clinically. To date, none of the available FP grading systems have gained widespread clinical acceptance. The work presented here describes the development and testing of a system for FP grading and assessment which is part of a comprehensive evaluation system for FP. The system is based on the Kinect v2 hardware and the accompanying software SDK 2.0 in extracting the real time facial landmarks and facial animation units (FAUs). The aim of this paper is to describe the development and testing of the FP assessment phase (first phase) of a larger comprehensive evaluation system of FP. The system includes two phases; FP assessment and FP classification. A dataset of 375 records from 13 unilateral FP patients was compiled for this study. The FP assessment includes three separate modules. One module is the symmetry assessment of both facial sides at rest and while performing five voluntary facial movements. Another module is responsible for recognizing the facial movements. The last module assesses the performance of each facial movement for both sides of the face depending on the involved FAUs. The study validates that the FAUs captured using the Kinect sensor can be processed and used to develop an effective tool for the automatic evaluation of FP. The developed FP grading system provides a detailed quantitative report and has significant advantages over the existing grading scales. It is fast, easy to use, user-independent, low cost, quantitative, and automated and hence it is suitable to be used as a clinical tool.

## 1. Introduction

Facial Paralysis (FP) is the partial loss of facial movements due to facial nerve pathology. Impairment of voluntary facial muscles innervated by the facial nerve, including muscles of facial expressions, leads to facial asymmetry [1]. FP is a very common condition. About 1.67% of people around the world can be affected by facial paralysis [2]. The early precise diagnosis and treatment can increase the chances of improvement and complete recovery at 3 to 9 months [3].

There is currently no standardized clinical assessment for lower motor impairment and most of the available grading tests are subjective, time consuming and not applied in routine daily practice. An accurate, fast, non-invasive, quantitative, and objective evaluation and classification system of FP is still required. Such a system is essential in selecting treatment and rehabilitation protocols as well as quantifying improvement in the follow-up phase.

Over the past sixty years, a large number of facial grading systems have been proposed and most of them are detailed in several comprehensive reviews [4–7]. The grading systems are divided into two categories; traditional grading scales and computer-assisted grading systems [5].

### 1.1. Traditional grading scales

Traditional grading scales are commonly used as ground truth in AI-based grading systems. Examples that are commonly used by clinicians include: the House-Brackmann Grading System (HBGS), the Facial Nerve Grading System 2.0 (FNGS 2.0), the Nottingham grading system, the eFace scale which assesses overall facial disfigurement [1] and several others [4,5]. Most of these grading systems are based on comparing distances between specific landmarks on the face at rest and during performing various facial exercises. These grading systems have several limitations. They are subjective and depend on experienced physicians [9]. They are time-consuming and labor-intensive since some require marking facial landmarks [5] and manually measuring the distances between them. In addition, most of the results evaluated using the traditional scales are not objective repeatable results.

### 1.2. Computerized grading systems

Computerized grading systems based on facial landmark detectors followed by machine learning and classification techniques overcome most of the limitations of traditional grading systems. Clearly, automatic and accurate facial landmarks localization is essential for the success of computerized applications such as facial recognition, facial expression detection, and facial paralysis classification [10]. Locations of facial landmarks reflect facial deformations and therefore can be used to create feature vectors for the face [11]. These vectors can be inputs to AI and machine learning algorithms for FP classification.

The review in [10] summarizes the available automatic facial landmarks identification techniques and reports some of the publicly available facial databases with landmarks. One example is a study of facial palsy detection based on 32 videos from YouTube from 22 FP patients [12]. It includes face detection, locating facial landmarks and identifying local affected palsy regions with labels Eyes or Mouth.

One study [13] validated the eFACE scale using video records of 83 FP patients. In this study, grading was performed by different individuals and eFACE scores demonstrated good intra-observer and inter-observer reliability, they were highly correlated with the traditional clinical grading systems but not with the Facial Disability Index. For more objective facial assessment, a machine learning-based automated assessment tool, Auto-eFACE, was developed and compared to eFACE. When tested on 160 photographs, Auto-eFACE differentiated normal faces from those with FP. However, Auto-eFACE gave lower scores than eFACE for identifying normal subjects [14].

Also based on the grading scales of eFace [8], another study [15] proposed using deep features of a pre-trained convolutional neural network (CNN) and training single linear repressors for each static and dynamic eFace sub-score. An annotated data set of 2D RGB images of 52 FP patients was used. Only static and dynamic eFace scores were considered. Static parameters are extracted from rest images while dynamic parameters are evaluated from images of facial movements. Synkinesis was not included as it depends on videos not 2D images. One limitation of this work is the subjective annotation of the data by one expert which leads to subjective predictions by the model.

Another study developed a Unity 3D application that can be used as a rehabilitation and grading system for FP [16]. In this study, the Kinect v2 was used to acquire the facial key points essential for evaluating facial symmetry. House-Brackmann methodology was then used to evaluate the FP degree. The FP evaluation in this study was later improved using the facial symmetry values as inputs to a neural network to classify between normal subjects and FP patients [17]. In addition, the normal side of the face was compared to the affected side according to the Nottingham grading system.

In another study, the symmetry of the face was examined in a small group of 11 FP patients [18]. After marking 50 reference points on the patient’s face, the face were scanned using Stereophotogrammetry to produce 3D images. Mirror^®^ Vectra software was then used to calculate point-to-point root mean square (RMS) for evaluating the symmetry index (SI). In this study, only the mouth movements were considered and the degree of FP was not reported.

In 2018, Guarin and Dusseldorp developed a software system based on machine learning techniques for automatic localization of facial key points and measurements of facial symmetry [19]. The software was developed based on a database of 2D facial images acquired from normal subjects. This may produce landmarks localization errors when tested on FP patients with large facial asymmetry. Another limitation is that the image resolution affects the accuracy, and also the head transformations like the yaw angle and tilt are not considered.

### 1.3. Limitations of computerized facial paralysis grading systems

Most of the current approaches cannot perform well with common existing challenging conditions [20]. One of these challenges is wearing accessories (e.g. glasses) and also the unusual appearance of the face (e.g. mustache, haircut …etc.). Furthermore, the existing systems suffer from large variations in the inter-personal results. This means that the subject is unable to perform the same expression uniformly all the time.

Several of the available studies [15,18] are based on small datasets with a lack in the disease diversity which limits the classification accuracy and makes it difficult to extend to large-scale applications.

Some of the current research use hand-crafted features methods in selecting the appropriate facial features in assessing FP [21,22]. This is may not be an optimal facial representation and can result in a low evaluation accuracy. Face capture using 2D imaging techniques may suffer from a series of limitations related to gestures, occlusion between extremities and lighting changes as well as the sensitivity to the external facial asymmetry caused by pose, orientation, illumination and shadows [23].

The systems based on Kinect 1.0 to capture the facial animation units (FAUs) suffer from some problems [24]. This is because only the 6 FAUs are unstable and not enough for upper and lower face features. Furthermore, lip corners as well as small variations in the features of the eyes could not be captured efficiently by Kinect 1.0.

For 3D facial movements capture, optical motion systems are used with placing specific reflecting markers on the subject’s face [25]. These systems are expensive and need a specialized clinician to put the markers in the correct places. Also these markers may disturb the patients and distort their facial movements.

Practically, the clinical evaluation of facial paralysis depends on the static facial asymmetry at the maximal movement and the dynamic change of movement. Most of the research focuses on just one aspect (the static asymmetry) in grading the FP.

### 1.4. Goals of the current research

The current research is part of a larger framework for designing, developing and testing a comprehensive evaluation system for FP using Artificial Intelligence (AI) and Machine learning (ML) algorithms. The system is based on the Kinect v2 and the SDK 2.0 for automatic extraction of facial landmarks and FAUs from FP patients. Feature vectors created from landmarks and FAUs are then used as inputs in the evaluation process. The goal of the system is to help the clinician assess the degree of impairment if not clinically detected, to provide a quantitative and objective grading system of FP statically and dynamically to assist in treatment decisions, to detect subtle improvement that cannot be detected clinically and finally to potentially predict the prognosis of different severity patterns.

The evaluation system consists of two phases; FP assessment and FP classification. The work presented in this paper focuses on the assessment phase and is an extension of previous research by the authors [26]. The previous study presented a system to classify five normal facial functions: smiling, eye closure, raising the eyebrows, blowing cheeks, and whistling as well as the rest state. The FP assessment phase described here includes three modules; (a) computing the symmetry index between both sides of the face while performing six facial movements, (b) determining the patient’s ability of performing each facial function and (c) grading the performance of each facial function of both sides separately. To the best of our knowledge, this comprehensive assessment has not been reported in the literature. The features created through these three modules will then fed to the FP classification phase (second phase) for better performance in classifying the FP severity level [27].

## 2. Materials and methods

**Fig 1:**
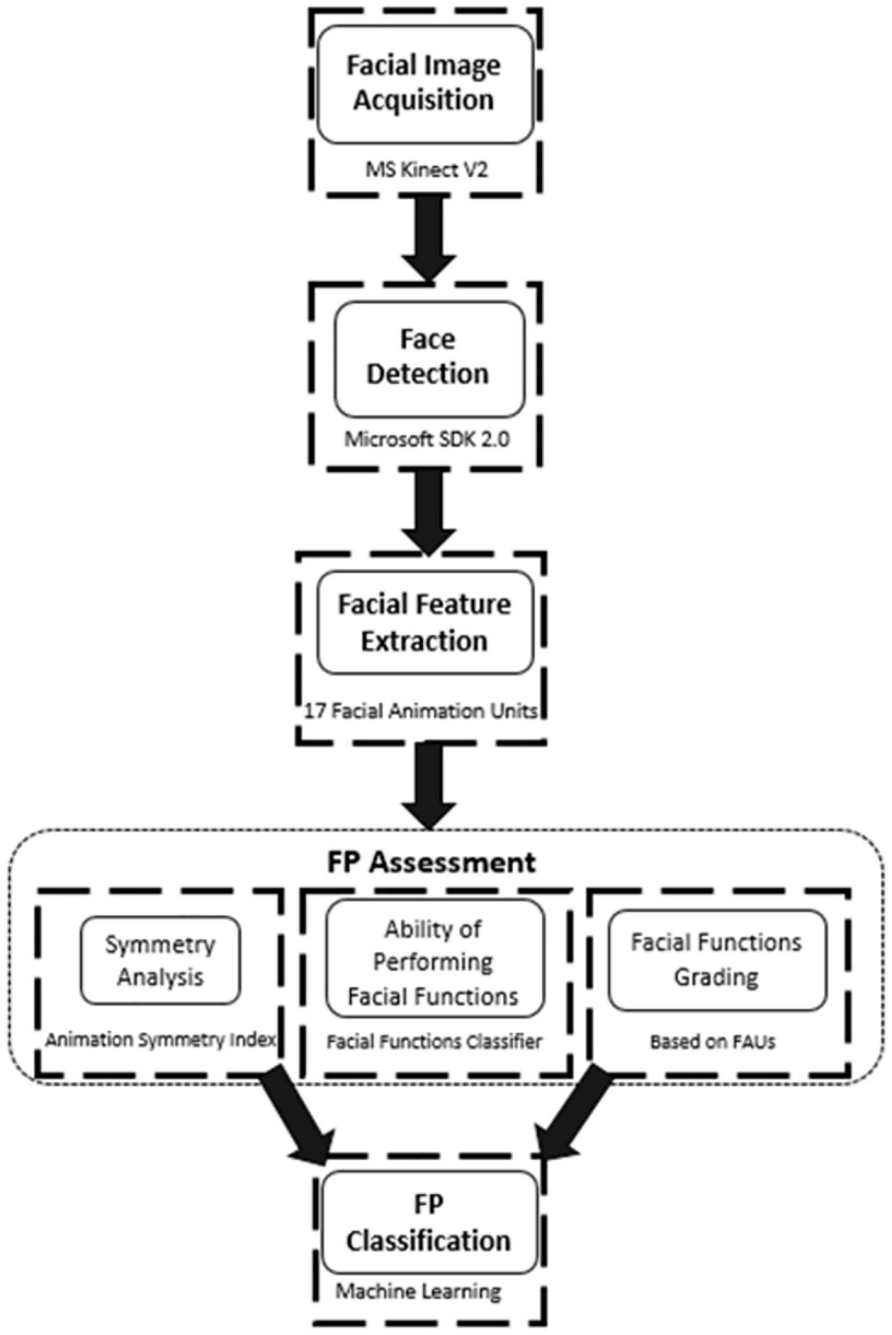
The block diagram of facial paralysis evaluation.

The complete block diagram of the FP evaluation procedure is shown in Fig 1. The research presented in this study is the automated FP assessment of six facial functions. This is performed using three separate modules which are described in detail in the following sections after data acquisition and feature extraction.

### 2.1. Data *acquisition*

Patients with different degrees of single-side FP were studied in Al Kasr El Aini hospital and Al Azhar hospital. The age of the patients ranges from thirteen to sixty years old, with 8 females and 5 males. Each patient sat on a seat about 50 cm high in a well-lit room at a distance of 1 m from the Kinect sensor. Each patient was asked to perform the 5 movements; raising eyebrows, closing eyes, smiling, blowing cheeks, and whistling. Several samples of each voluntary movement besides the rest state were acquired. There was enough resting time between the acquired samples from the same subject. In total, the dataset contains 375 records from 13 patients. Patients and parents of minor patients were informed of the research procedures and signed an informed consent form.

### 2.2. Feature extraction

Facial Action Coding System (FACS) is a system of describing the movements of facial muscles and how these movements reflect changes in the face’s appearance [28]. Some appearance changes are the outcome of movements of multiple muscles and some muscles can have more than one action. FACS analyzes each facial expression into separate components of facial muscle movement, named action units (AUs) [29]. The Kinect V2 with the SDK 2.0 not only provide a library for automatically acquiring 3D facial landmarks but also the Facial Animation Units (FAUs) which reflect the action units (AUs). Landmarks and FAUs of the face have been used previously in facial movements’ evaluation [16,17,30–33] facial emotion and expression recognition [34–36], and in facial paralysis evaluation [37].

In this study, the seventeen FAUs (in Table 1) were tracked for each facial movement by the Kinect sensor and the SDK 2.0. These features were then processed for FP evaluation.

**Table 1:** Seventeen Facial Animation units (FAUs) Extracted using Kinect SDK 2.0 [26].

Facial Animation unit	Descriptor
FAU1	Jaw Open
FAU2	Lip Pucker
FAU3	Jaw Slide Right
FAU4	Lip Stretcher Right
FAU5	Lip Stretcher Left
FAU6	Lip Corner Puller Left
FAU7	Lip Corner Puller Right
FAU8	Lip Corner Depressor Left
FAU9	Lip Corner Depressor Right
FAU10	Left Cheek Puff
FAU11	Right Cheek Puff
FAU12	Left Eye Closed
FAU13	Right Eye Closed
FAU14	Right Eyebrow Lowerer
FAU15	Left Eyebrow Lowerer
FAU16	Lower Lip Depressor Left
FAU17	Lower Lip Depressor Right

### 2.3. Symmetry analysis

FP patients have resting facial asymmetry and are also unable to move the facial muscles of both sides correctly and symmetrically. It is therefore important to evaluate the asymmetry of the face for various facial movements (dynamic asymmetry) in addition to the resting state asymmetry (static asymmetry).

The FAUs are acquired using the Kinect sensor from each subject while performing the 6 movements. In this module, the values of FAUs are compared between right and left sides of the face during the 6 movements to compute the animation symmetry index (ASI) of specific areas of the face. The ASI defined here is a single value which quantifies the symmetry of each facial feature between both sides.

The ASI was assessed for three different areas; eyebrows symmetry (ASI_Eyebrows), eyes symmetry (ASI_Eye), and mouth symmetry (ASI_Mouth). Twelve of the seventeen FAUs were used for symmetry assessment as shown in Table 2.

**Table 2:** The twelve FAUs used for symmetry analysis.

Facial Area	FAUs
Eyes	FAU2: Left Eye Closed FAU3: Right Eye Closed
Eyebrows	FAU4: Left Eyebrow Lowerer FAU5: Right Eyebrow Lowerer
Mouth	FAU6: Lip Corner Puller Left FAU7: Lip Corner Puller Right FAU8: Lip Stretcher Left FAU9: Lip Stretcher Right FAU10: Lip Corner Depressor Left FAU11: Lip Corner Depressor Right FAU16: Left Cheek Puff FAU17: Right Cheek Puff

The ASI of the eyebrows is computed from the absolute difference between the two FAUs; FAU4 (Left Eyebrow Lowerer) and FAU5 (Right Eyebrow Lowerer) subtracted from 1 then multiplied by 100 as shown in Eq (1) to indicate the percentage of symmetry between right and left eyebrows.


$$ASI\_Eyebrows\left(\%\right)=\left(1-\left|FAU4-FAU5\right|\right)*100\%$$ (1)

The ASI of the eyes is calculated using a similar equation but with the FAUs of the eyes; FAU2 (Left Eye Closed) and FAU3 (Right Eye Closed) as shown in Eq (2).


$$ASI\_Eyes\;\left(\%\right)\;=\left(1-\left|FAU2-FAU3\right|\right)*100\%$$ (2)

However the ASI of the mouth depends on 8 FAUs; FAU6, FAU7, FAU8, FAU9, FAU10, FAU11, FAU16, and FAU17. Four types of symmetry were evaluated then averaged to reflect the ASI of the mouth.

First, the lip corner puller symmetry is calculated from FAU6 (Lip Corner Puller Left), and FAU7 (Lip Corner Puller Right) through Eq (3). Second, the lip stretcher symmetry is calculated between both sides from FAU8 (Lip Stretcher Left), and FAU9 (Lip Stretcher Right) through Eq (4). Then the lip corner depressor symmetry is calculated from FAU10 (Lip Corner Depressor Left), and FAU11 (Lip Corner Depressor Right) through Eq (5). And finally, the cheeks symmetry is calculated between both sides from FAU16 (Left Cheek Puff), and FAU17 (Right Cheek Puff) through Eq (6). These ASIs related to the mouth are then averaged to indicate the percentage of the symmetry of the mouth as shown in Eq (7).


$$ASI\_LipPuller\;\left(\%\right)=\left(1-\left|FAU6-FAU7\right|\right)*100\%$$ (3)

$$ASI\_LipStretcher\;\left(\%\right)\;=\left(1-\left|FAU8-FAU9\right|\right)*100\%$$ (4)

$$ASI\_LipDepressor\;\left(\%\right)\;=\left(1-\left|FAU10-FAU11\right|\right)*100\%$$ (5)

$$ASI\_Cheeks\;\left(\%\right)\;=\left(1-\left|FAU16-FAU17\right|\right)*100\%$$ (6)

$$ASI\_Mouth\;\left(\%\right)=\;\frac{ASI\_LipPuller\;+ASI\_LipStretcher\;+\;ASI\_LipDepressor\;+\;ASI\_Cheeks}{4}\%$$ (7)

The ASIs of the three facial regions; eyes, eyebrows, and mouth were calculated for each of the 6 movements; rest, eye closure, raising eyebrows, smiling, blowing cheeks, and whistling. The total ASI for each facial movement can be calculated by averaging the symmetry of the three facial areas; eyes, eyebrows, and mouth as shown in Eq (8).


$$Total\_ASI\;\left(\%\right)=\;\frac{ASI\_Eyes+ASI\_Eyebrows+ASI\_Mouth}{3}\;\%$$ (8)

### 2.4. Ability of performing facial functions

This module of FP assessment determines if the patient was able to perform each required facial movement. This is based on the SVM classifier previously developed by the authors and trained on normal subjects [26]. The nine most important differences in FAUs, as previously discussed in [26], were acquired from the FP patients while performing the 6 facial movements and used in the classification.

The result of the classifier shows if the facial movement is actually done or not. If the movement is correctly classified, the result will be “Yes”. While if the movement is classified with another movement type, the result will be “No”. When developing this module, a third result value “may be” was added. This third result is assigned when the confidence value of the probability estimate of the classified movement is very near to another movement with just less than 7% difference. This threshold value was manually specified. For example, if the classifier was 40% sure for smiling, and 46% sure for closing eyes, then the output regarding these two movements will be “may be” while the output regarding the other movements will be “No”.

This phase of assessment only shows whether the patient can move the muscles corresponding to each specific facial movement or not. It does not specify the power of muscles’ movements and the facial asymmetry between the two sides in performing the movement. This means that the movement may be classified correctly while the patient was not performing it in the normal manner. Although this phase of grading FP is important, it is not sufficiently precise, therefore more analysis in grading facial functions performance was required. Advanced facial functions grading for both sides is discussed in the following section.

### 2.5. Facial functions grading

In this module, certain portions of the face were graded while performing the selected facial movements; smiling, closing the eyes, lifting the eyebrows, blowing cheeks, and whistling.

Some of these voluntary movements are considered in traditional standard scales however in this system they are evaluated using a more accurate approach and the resultant report has more grading details. Assessment of each movement is performed for each side of the face independently. The system automatically calculates the percentage of achieving a certain voluntary movement (i.e. rate of movement) successfully and then assigns a grade for each side separately.

#### 2.5.1. Defining the most affected FAUs in each movement

In different facial movements, each FAU has different degrees of involvement. For example, the change of FAU2 (Left Eye Closed) and FAU3 (Right Eye Closed) from their rest values reflects how much the subject closed his eyes. Each movement’s performance of both sides of the face is graded separately depending on the FAUs affecting the movement. Therefore, it is necessary to determine the most affected FAUs involved in each movement.

**Fig 2:**
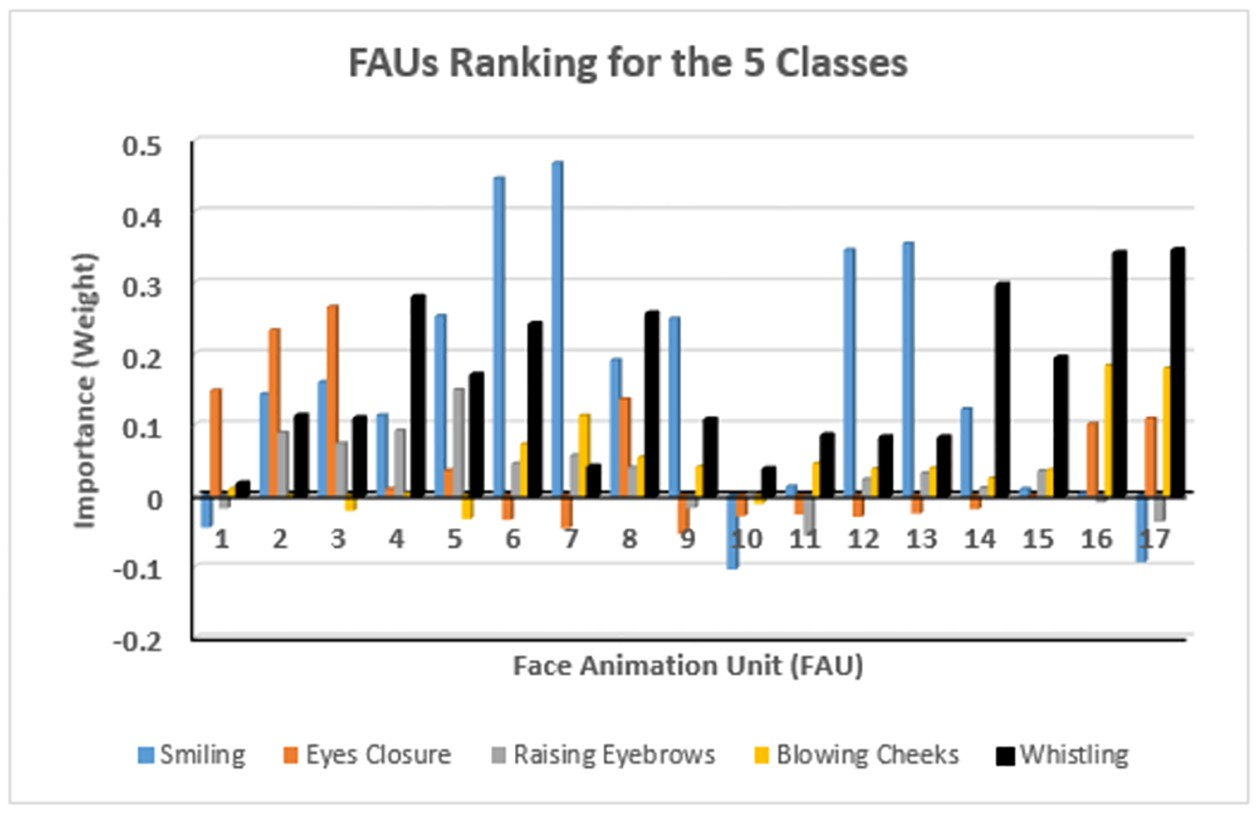
Feature ranking applied on the 17 differences in FAUs in the 5 facial movements; smiling, eye closure, raising eyebrows, blowing cheeks, and whistling.

A feature ranking tool was used in selecting the most important features that affect the classification of the facial movement with respect to the rest state. Relief-based feature selection algorithm [26] with 10 nearest neighbors was applied on the seventeen difference in FAUs between one of the five classes and the rest class. The algorithm was applied on the normal data set developed [26]. Each feature and its importance (weight) in performing each facial movement are shown in Fig 2. Table 3 summarizes the most affected FAUs involved in each facial movement.

**Table 3:** The most affected FAUs in each facial movement.

Facial Movement	FAUs
Smiling	FAU6: Lip Corner Puller Left FAU7: Lip Corner Puller Right
Eye Closure	FAU2: Left Eye Closed FAU3: Right Eye Closed
Raising Eyebrows	FAU4: Left Eyebrow Lowerer FAU5: Right Eyebrow Lowerer
Blowing Cheeks	FAU16: Left Cheek Puff FAU17: Right Cheek Puff
Whistling	FAU14: Lip Pucker FAU16: Left Cheek Puff FAU17: Right Cheek Puff

Note that in Table 3, the changes of values in FAU16 and FAU17 are involved in the two movements; blowing cheeks and whistling. In blowing cheeks, the values of FAU16 and FAU17 are increased from their rest values while in whistling, the values of FAU16 and FAU17 are decreased from their rest values.

#### 2.5.2. Grading criteria

Once the FAUs of the selected frame are collected, the analysis and calculations are performed to quantify the change of FAUs values between a given frame and the rest frame. For example, when assessing the eye function, the system automatically acquires the eyes’ FAUs; FAU2 (Left Eye Closed) and FAU3 (Right Eye Closed) in two different frames. The first frame is the resting frame and is captured with no movements of both eyes while the second frame is captured when the patient is asked to close his eyes. The ability of the patient to close his eyes completely is evaluated from the difference between the FAUs (associated with the eye closure) in the two frames.

*A*) *Smiling*

The smiling voluntary movement is essential in assessing the mouth function. Evaluating the performance of smiling is based on the FAU6 and FAU7 as shown in Table 3. The system detects that the patient is smiling if there is a significant difference in the values FAU6 and FAU7 from the rest. How much the patient is smiling is calculated from Eqs (9) and (10) for the left and right sides respectively.


$$Delta\_Smiling\_L\left(\Delta \;\text{FAU}6\right)=\text{FAU}6{\left|{}_{\text{smiling}}-\text{FAU}6\right|}_{\text{rest}}$$ (9)

$$Delta\_Smiling\_R\;(\Delta \;\text{FAU}7)=\text{FAU}7{\left|{}_{\text{smiling}}–\text{FAU}7\right|}_{\text{rest}}$$ (10)

A score of 1 is assigned for each 0.1 increase in the FAU’s value with a maximum of 10/10 when the difference reaches its maximum value of 1. The resultant report shows whether each side of the mouth has moved while smiling or not and to what score if it has moved.

*B*) *Eye Closure*

The same method is used for grading the eye closure movement but using its corresponding FAUs; FAU2 and FAU3 as shown in Table 3. Assessing the performance of closing the eyes depends on the change in the FAU2 and FAU3 values between the two frames; the rest frame and the frame captured while closing the eyes. How much the eye is closed is calculated for both left and right sides independently from Eqs (11) and (12) respectively.


$$Delta\_Eye\_L\;(\Delta \text{FAU}2)=\text{FAU}2{\left|{}_{\text{Eye}\;\text{Closure}}–\text{FAU}2\right|}_{\text{rest}}$$ (11)

$$Delta\_Eye\_R\;(\Delta \text{FAU}3)=\text{FAU}3{\left|{}_{\text{Eye}\;\text{Closure}}–\;\text{FAU}3\right|}_{\text{rest}}$$ (12)

*C*) *Raising Eyebrows*

When lifting the eyebrows, the values of FAU4 and FAU5 will be decreased from their rest values. The capability of the patients to raise the eyebrows is calculated for both left and right sides independently from Eqs (13) and (14) respectively.


$$Delta\_Eyebrows\_L\left(\Delta \;\text{FAU}4\right)={\left|(\text{FAU}4\right|}_{\text{Raising}\;\text{Eyebrows}}–\text{FAU}4\left|{}_{\text{rest}})\right|$$ (13)

$$Delta\_Eyebrows\_R\left(\Delta \;\text{FAU}5\right)={\left|(\text{FAU}5\right|}_{\text{Raising}\;\text{Eyebrows}}–\text{FAU}5\left|{}_{\text{rest}})\right|$$ (14)

A grade of 1 is assigned for each 0.1 decrease in the FAU’s value from the rest value.

*D*) *Blowing Cheeks*

A similar criteria is used for assessing blowing cheeks but using its corresponding FAUs; FAU16 and FAU17 as shown in Table 3. The power of blowing cheeks is calculated for both left and right sides independently from Eqs (15) and (16) respectively.


$$Delta\_Cheeks\_L\left(\Delta \;\text{FAU}16\right)=\text{FAU}16{\left|{}_{\text{Blowing}\;\text{Cheeks}}–\text{FAU}16\right|}_{\text{rest}}$$ (15)

$$Delta\_Cheeks\_R\left(\Delta \;\text{FAU1}7\right)=\text{FAU}17{\left|{}_{\text{Blowing}\;\text{Cheeks}}–\text{FAU}17\right|}_{\text{rest}}$$ (16)

*E*) *Whistling*

When performing the whistling movement, the value of FAU14 is increased. The increase in FAU14’s value reflects the power of whistling. Also, the values of FAU16 and FAU5 are decreased from their rest values. The score of whistling performance is calculated from Eqs (17), (18) and (19).


$$Delta\_Whistling\left(\Delta \text{FAU}14\right)=\text{FAU}14{\left|{}_{\text{Whistling}}–\text{FAU}14\right|}_{\text{rest}}$$ (17)

$$Delta\_Whistling\_L\left(\Delta \;\text{FAU}16\right)={\left|(\text{FAU}16\right|}_{\text{Whistling}}–\text{FAU}16\left|{}_{\text{rest}})\right|$$ (18)

$$Delta\_Whistling\_R\left(\Delta \;\text{FAU}17\right)={\left|(\text{FAU}17\right|}_{\text{Whistling}}–\text{FAU}17\left|{}_{\text{rest}})\right|$$ (19)

## 3. Results

The modules of FP assessment were tested and evaluated as described in the following subsections.

### 3.1. Symmetry analysis

The first module “Symmetry analysis” in the FP assessment stage computes the ASIs of each area of the face (eyes, eyebrows, mouth) statically (in the rest state) and dynamically (in the 5 movements; smiling, closing eyes, raising eyebrows, blowing cheeks, and whistling). The module was tested and evaluated on the thirteen FP patients. Samples of the results on 9 FP patients are shown in Table 4. As seen in Table 4, a report for each patient would include these 18 values of ASI’s.

**Table 4:** Animation symmetry indices (ASIs) calculated from 9 FP patients.

Patient #	Animation Symmetry Index	Facial Movement					
		Rest	Smiling	Closing Eyes	Raising Eyebrows	Blowing Cheeks	Whistling
**#1**	**Eyes %**	94	90	84	97	97	83
	**Eyebrows %**	90	91	87	96	91	97
	**Mouth %**	80	86	85	82	83	62
**#2**	**Eyes %**	86	92	92	82	91	97
	**Eyebrows %**	90	93	89	83	95	97
	**Mouth %**	83	92	90	80	88	85
**#3**	**Eyes %**	83	93	90	89	94	96
	**Eyebrows %**	88	92	92	91	94	95
	**Mouth %**	90	86	87	84	92	91
**#4**	**Eyes %**	97	93	69	92	93	81
	**Eyebrows %**	92	90	89	95	88	97
	**Mouth %**	92	94	92	91	92	87
**#5**	**Eyes %**	83	87	71	90	81	85
	**Eyebrows %**	87	95	85	95	88	82
	**Mouth %**	90	93	90	90	74	91
**#6**	**Eyes %**	87	93	36	94	85	94
	**Eyebrows %**	88	87	71	93	83	93
	**Mouth %**	82	73	77	84	89	77
**#7**	**Eyes %**	98	96	95	97	88	95
	**Eyebrows %**	92	94	89	97	88	88
	**Mouth %**	91	84	90	89	91	91
**#8**	**Eyes %**	88	89	89	88	77	88
	**Eyebrows %**	94	96	96	86	91	81
	**Mouth %**	93	78	89	92	77	77
**#9**	**Eyes %**	91	86	87	81	95	89
	**Eyebrows %**	98	96	95	96	76	91
	**Mouth %**	83	85	86	86	80	89

### 3.2. Ability of performing facial functions

The second module of FP assessment is called “ability of performing facial functions”. It uses the facial function classifier introduced in [26] to determine if the patient can perform specific facial functions. The confidence value of the probability estimates for each movement resulted from the facial function classifier beside the classified result are used to assign one of the three values “Yes”, “No”, “may be” as previously discussed in section 2.4. This module was tested on 13 palsy-affected subjects. Samples of the results on 9 FP patients are shown in Table 5. For each facial movement, it is shown if the patient can perform the movement or not.

**Table 5:** Facial functions evaluation (9 FP patients).

Patient (L/R)		Ability of performing the facial movement				
		Smiling	Closing Eyes	Raising Eyebrows	Blowing Cheeks	Whistling
patient1 (L)	Done?	Yes	Yes	Yes	Yes	No
	Left	6	6	3	3	0
	Right	8	5	4	0	0
patient2 (L)	Done?	Yes	Yes	Yes	Yes	No
	Left	4	5	2	2	0
	Right	3	3	3	0	0
patient3 (R)	Done?	No	No	No	Yes	No
	Left	2	3	7	2	0
	Right	3	2	8	2	0
patient4 (R)	Done?	No	Yes	Yes	Yes	No
	Left	0	6	2	2	0
	Right	0	7	2	3	0
patient5 (R)	Done?	Yes	Yes	Yes	May be	No
	Left	2	5	4	0	0
	Right	3	4	3	0	0
patient6 (R)	Done?	Yes	Yes	Yes	Yes	May be
	Left	4	3	2	0	1
	Right	6	2	3	1	0
patient7 (L)	Done?	May be	Yes	Yes	Yes	Yes
	Left	3	3	4	2	1
	Right	0	4	3	1	2
patient8 (R)	Done?	Yes	Yes	No	Yes	May be
	Left	6	2	0	3	1
	Right	6	4	0	5	0
patient9 (R)	Done?	Yes	Yes	Yes	Yes	No
	Left	4	5	3	1	0
	Right	6	5	3	2	0

### 3.3. Facial functions grading

The third module of FP assessment is called “facial functions grading”. It computes a score for the degree of the facial function performance in both sides of the face separately. The module was tested on 13 palsy-affected patients. The results of 9 FP patients are summarized in Table 5. For each facial movement, it is determined whether the patient can perform the movement or not and also with what grade it is performed for each of the left and right sides separately. The grade takes the value in the range from 0 to 10 indicating the grade of movement.

**Fig 3:**
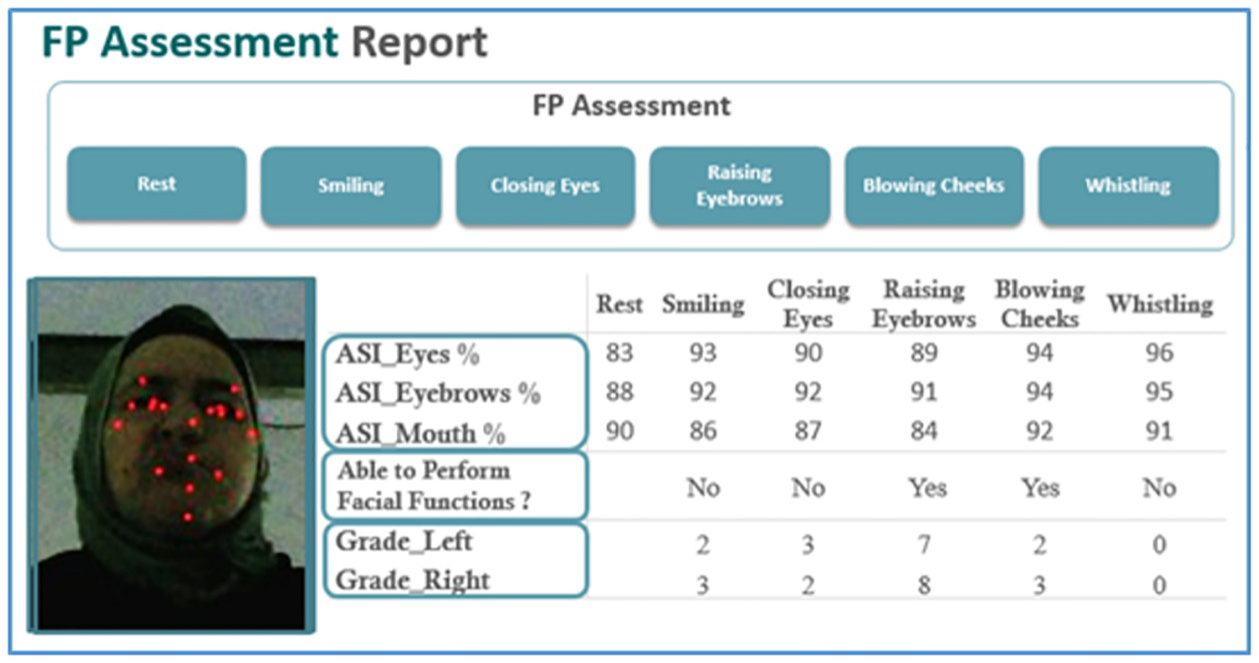
FP assessment report.

The system provides a report (as in Fig 3) for each patient including the following:

- Eighteen values of ASI’s. Each ASI is calculated for one of the three areas of the face; eyes, eyebrows, and mouth while doing one of the five facial movements in addition to the rest state (as shown in Table 4).
- Five Boolean values for the five facial functions determining if the patient can perform specific facial functions. The Boolean value takes one of the three values “Yes”, “No”, “may be” as shown in Table 5.
- Ten grading values reflecting the facial function performances. Each grade is calculated for each side of the face independently while doing one of the five facial movements (as shown in Table 5). The grade value ranges from 0 to 10 specifying the power of movement.

## 4. Discussion

Although there have been many advances in computerized FP grading systems [38,39], a comprehensive, quantitative, objective, and precise system is still required. The aim of the current research is designing, developing and testing a comprehensive evaluation system for FP including automatic facial landmark detection, assessment and classification. The work presented in this paper is an extension of previous research by the authors [26]. The previous study presented a system to classify five normal facial functions: smiling, eye closure, raising the eyebrows, blowing cheeks, and whistling as well as the rest state. The work described here is the development and testing of three assessment modules for FP patients; symmetry analysis, ability of performing facial functions, and facial function grading.

The first step of the work was selecting the method of facial data acquisition. The majority of previous work related to FP assessment used two-dimensional dataset images which are affected by the orientation and lighting [23]. In addition, automatic landmark detection has been performed on 2D video images [12] or depends on manual and thus subjective landmark detection [18,25]. For 3D facial capture, optical systems have been previously used [25]. However, these systems are expensive and need a specialized clinician to put markers in the correct places on the face. These markers may disturb the patients and distort their facial movements. The old version of the Kinect 1.0 has also been tested and previous research highlighted that the six FAUs provided by it are not stable and are insufficient for representing the upper and lower facial features [24]. Furthermore, the subtle variations in the eyes area and other points such as lip corners could not be detected correctly.

The Kinect V2, with SDK 2.0, overcomes several of these limitations for 3D facial data acquisition. It is automatic, fast, accurate and eliminates the need for a specialized clinician or additional feature recognition software. The system uses images of color and depth to extract FAUs and 3D landmarks, and the data shows high performance even with unusual appearance of the face such as mustache or wearing accessories (e.g. glasses) unlike with other systems [20]. Also there is no need for markers on the face and hence no physical contact with the patient which is an advantage during the Covid-19 pandemic. Furthermore, the FAUs reflect the action units (AUs) which in turn separate facial expressions into separate components of facial muscle movement. Thus the FAUs were selected as a viable option for features in FP assessment and classification in this work. However, this posed the first challenge faced which was the unavailability of FP datasets with the FAUs as features. Second, nothing of the available research is based on similar methods to compare the results with.

The asymmetry between the left and right facial sides is essential in evaluating FP as suggested before [16,17,40,41]. In addition, symmetry analysis is an important step after facial reanimation treatment to evaluate the success of surgical intervention [18]. Most of the prior work targets only the static asymmetrical facial features and do not consider the dynamic change of features during certain facial movements.

In this study, the ASI was computed for three areas of the face statically (in the rest state) and dynamically (in the 5 movements; smiling, closing eyes, raising eyebrows, blowing cheeks, and whistling). The results of testing on FP patients are shown in Table 4, and a detailed report for each patient would include these 18 values of ASI’s. The results show that for most of the patients, the ASI of the mouth was smaller (indicating more asymmetry) than for the eyes and eyebrows. This is possibly because, according to the clinician, the area of the mouth is usually the area which recovers more slowly than the rest of the face and thus remains more asymmetrical for a longer time.

In different facial movements, each FAU has different degrees of involvement. Thus in order to use the FAUs to classify and grade the different facial movements it was necessary to determine which FAUs were most involved in causing each movement. A feature ranking tool was used in selecting the most important features that affect the classification of the facial movement with respect to the rest state. Relief-based feature selection algorithm [26] with 10 nearest neighbors was applied on the seventeen difference in FAUs between one of the five classes and the rest class. Each feature and its importance (weight) in performing each facial movement are shown in Fig 3. Table 3 summarizes the most affected FAUs involved in each facial movement.

The results of the second and third modules are shown in Table 5. According to the clinician, a patient with unilateral FP cannot perform most of the movements with the affected side while he/she is able to achieve the movements by the healthy side. These movements are smiling, closing the eyes, raising the eyebrows, and whistling. On the other hand, he/she can blow the cheek of the affected side but cannot blow the unaffected cheek. Hence, patient 1 and patient 2 with the left-side FP could not blow their right cheek (as shown in Table 5) while blowing their left ones.

According to the visual assessment by the physician, most of the patients could not perform the lip puckering in whistling movement. They may be able to expand the mouth in smiling but it is hard to shrink their mouth to achieve whistling. As noted in Table 5, most of the patients could not achieve the whistling movement and only one patient (patient7) could perform the movement (confirmed by the classifier) with a very low degree for both left and right sides.

Although patient 3 (see Table 5) performs well in raising his/her eyebrows for the left and right sides with grades 7 and 8 respectively, the facial function classifier detects that he/she does not raise the eyebrows. This may be due to several reasons. First, the asymmetrical facial features between both sides may affect the classification result. Second, the involuntary movement of other facial features due to the level of paralysis disperse the result. And last, it may be due to the misclassification rate of the classifier. As previously discussed in [26], the maximum performance of the facial function classifier was (Accuracy = 96.7%, Sensitivity = 90.2%, and Specificity = 98%) with 3.3% misclassification rate.

Also, grading the facial functions (Table 5) shows that patient 5 could not blow his/her cheeks. However the facial function classifier does not make sure that they could not do these movements. The confidence value of the probability estimates for the movements are not low enough to confirm the decision of “No”.

The limitation in this work is testing on a small number of FP patients and the unavailability of some severity levels of FP. This is a limitation also reported in other work [15,18]. The most common causes of FP are influenza, cold, or other upper respiratory ailments. Therefore the number of FP patients increases in the beginning of the winter season and this number decreases again the rest of the year. In addition, the Covid-19 pandemic limited the number of FP patients visiting hospitals and clinics during the past two years. More FP patients with various FP severity levels should be considered in future work.

The work presented in this paper to the best of our knowledge is unique in providing a comprehensive assessment of FP which includes; automatic detection of facial features followed by quantitative static and dynamic symmetrical analysis of the face and grading the performance of achieving the facial functions for both sides individually. The procedure is not painful or invasive and does not involve any discomfort.

This study has some significant contributions. First, building a facial palsy dataset of 375 records from 13 FP patients performing the 6 facial movements. There is no similar dataset publicly available with the FAUs features. Second, a robust approach for comprehensive assessment of FP was introduced. It does not depend only on the static asymmetry of the face but also the dynamic evaluation of facial symmetry was considered. Third, validating that the FAUs (captured automatically by the Kinect v2 sensor) can be used in FP assessment.

## 5. Conclusion

The work in this paper is a part of a comprehensive evaluation system of facial paralysis. The evaluation system consists of two stages; FP assessment and FP classification. This paper describes the development of the FP assessment stage. Three different modules were developed; symmetry analysis, ability of performing facial functions, and facial functions grading.

The module of symmetry analysis is responsible for evaluating the static and dynamic symmetry indices between both facial sides in each individual facial movement. The second module “ability of performing facial functions” detects if the patient can perform the required function or not. This module depends on the facial function classifier previously developed by the authors [26] and trained on normal subjects performing the 6 facial functions. The third module “facial functions grading” evaluates the power of each facial movement in each facial side individually based on the affected facial features.

Our study validates that the seventeen FAUs acquired using the Kinect v2 can be used in evaluating facial paralysis. The developed system is fast, accurate, non-invasive, objective, and provides detailed quantitative results.

There is ongoing work on FP classification. The FP classifier is based on machine learning algorithms to classify different severity levels of facial paralysis.

The work presented in this study can be extended to be a facial virtual rehabilitation tool. Enhancing the patient’s engagement with 3D virtual environments and games can improve progress. It can be self-adaptive and customized to target specific disabilities. Based on the animation units (AUs) that can be acquired from facial images, a mobile facial assessment application can be developed. The AUs can be extracted from mobile images using AI and image processing techniques.

## Supporting information

S1 Dataset (XLSX)
